# Machine learning of factors for improving oyster hatchery production

**DOI:** 10.1371/journal.pone.0345084

**Published:** 2026-03-20

**Authors:** Srishti Vishwakarma, Matthew W. Gray, Greg M. Silsbe, Vyacheslav Lyubchich

**Affiliations:** 1 Computational Sciences & Engineering Division, Oak Ridge National Laboratory, Oak Ridge, Tennessee, United States of America; 2 Horn Point Laboratory, University of Maryland Center for Environmental Science, Cambridge, Maryland, United States of America; 3 Chesapeake Biological Laboratory, University of Maryland Center for Environmental Science, Solomons, Maryland, United States of America; Bangladesh Agricultural University, BANGLADESH

## Abstract

Oyster aquaculture and restoration in the Chesapeake Bay are vital, yet hatcheries frequently struggle with inconsistent larval growth and sudden mass mortality events. Unpredictable disruptions in larval production cause large economic losses, represent a perceived risk to growers, and impede industry expansion. To better understand associations between production yield and its potential predictors, we applied machine learning (random forest, and neural network) and statistical (generalized additive model) models to a comprehensive dataset of environmental, water quality, and operational parameters from a Maryland oyster hatchery, aiming to identify key yield predictors and develop a robust forecasting tool. We used recursive Boruta algorithm for variable selection, pinpointing critical predictors, and employed cross-validation to fine-tune model settings. Shapley value analysis offered crucial insights into model interpretations, highlighting week number, Normalized Difference Vegetation Index, salinity, turbidity, and fecundity as primary drivers of yield variability. For low-yield cases, salinity-related variables were particularly important. Our findings provide an early warning system for potential production downturns, empowering hatchery operators to make data-driven decisions for optimizing water conditions, feeding schedules, and broodstock management. By boosting predictability and efficiency, this research directly supports economic stability of the oyster industry and ecological health of the Chesapeake Bay.

## Introduction

The sustained success of oyster aquaculture, along with critical fisheries augmentation and restoration efforts in the Chesapeake Bay, fundamentally depends on reliable hatchery production of oyster larvae. Despite the dedicated expertise of the skilled hatchery staff, shellfish hatcheries often face periods of suboptimal larval growth and inconsistent production levels. The Horn Point Laboratory Oyster Hatchery (HPLOH) exemplifies this variability, with average yield rates fluctuating between 5% and 17% and occasional exceptional cases exceeding 50% yield [1].

In addition to chronic variability, HPLOH and other hatcheries commonly contend with acute mortality events, colloquially known as “crashes” [2,3]. Beyond microbial contamination [4], abrupt fluctuations in ambient water quality–such as harmful algal blooms or sudden changes in salinity or oxygen levels–can also profoundly disrupt culture conditions and critical components of the production process, including broodstock conditioning or the health of algae cultures essential for larval feed [3].

To mitigate these challenges and maintain suitable production conditions, hatcheries routinely monitor the quality of their source water alongside production metrics. When suboptimal conditions are detected, facilities can implement immediate adjustments, such as salinity modifications, to counteract adverse environmental effects and restart production cycles. However, the root causes of persistent production inefficiencies remain elusive. Hatchery staff typically operate under time and resource constraints that preclude exhaustive investigations into these underlying issues. Consequently, the prevailing approach to managing low-yield cases often involves flushing compromised water, meticulously cleaning the tanks, and hoping that they are refilling with “good” water that will allow production to resume. Unfortunately, adverse environmental conditions can persist for extended periods [1,5], severely curtailing regional oyster production and inflicting substantial financial losses on the industry (e.g., over $110 million during the “Seed Crisis” in the Pacific Northwest [6]). Therefore, there is a critical need to identify robust predictors of hatchery yield, particularly to anticipate and prevent costly low-yield events.

Addressing this critical gap, our study introduced an innovative approach by applying data-driven machine-learning methods to enhance the resilience and efficiency of oyster production. We leveraged these methods to transform complex environmental and biological data into actionable insights. By doing so, we aimed to provide tools that will not only enhance production reliability, thereby increasing the profitability of both public and private hatcheries through improved resource utilization and waste minimization, but also ensure a more stable seed supply. A reliable seed supply is paramount for alleviating concerns among oyster growers in Maryland and beyond, ultimately fostering greater opportunities for industry expansion and contributing to the economic well-being of local communities that are reliant on the oyster industry. This research contributes to improving the health of the Chesapeake Bay by supporting its keystone species and preserving a vital part of Maryland’s cultural heritage.

## Data

### Hatchery data

Our study utilized a comprehensive dataset combining long-term hatchery production records from [1] with more recent data from the HPLOH. This combined dataset encompasses the years 2011–2021, providing detailed information pertaining to broodstock, spawning, and larval production within the HPLOH.

The primary focus of our investigation was the hatchery yield, defined as the percentage of pediveliger oyster larvae (i.e., eyed larvae) successfully produced relative to the total number of fertilized eggs initially introduced into a tank:


$$\text{Yield}=\frac{\text{Eyed larvae produced}}{\text{Eggs added to tank}}\times 100\%.$$ (1)

To ensure a precise understanding of the factors influencing production outcomes, our analysis exclusively focused on larvae that were not mixed during the production process. Consequently, data from mixed batches were excluded from this study.

Data cleaning and preparation steps for the hatchery yield included:

- All yields were assigned a value of zero if the HPLOH records indicated that the batch was “dumped” due to a production crash.
- Records with HPLOH notes explicitly stating “no eggs” (7 records) or those lacking information on the number of eyed larvae (9 records) were removed from the dataset.
- Records exhibiting an unusually short production rate (defined as the number of days until the first eyed oysters were observed) were excluded. We identified three such records with production rates below 9 days, which typically suggest an accelerated production schedule for specific larvae buyers, potentially deviating from the standard HPLOH protocols.

From the HPLOH records, we identified several potential predictors of yield across different stages of the oyster production process:

1. **Conditioning:**
  - Average conditioning pH (NBS units)
  - Average conditioning salinity (PSU)
  - Average conditioning temperature (^°^C)
2. **Spawning:**
  - Average shell height of females (mm)
  - Average shell height of males (mm)
  - Fecundity (millions of eggs per female)
  - Gonadal index (scale of 1–4)
3. **Larval culture:**
  - Week of the year when production commenced (1–52)

Our selection of predictors was more focused compared to [1], prioritizing variables that could explain and predict yields before the production process began. For example, the year of broodstock collection was excluded due to its limited predictive utility, and the percentage survival to the prodissoconch II stage was not used as this information becomes available only later in the production cycle.

To establish a consistent reference point for environmental data integration, the date when each new larvae tank was filled with ambient water was calculated using the HPLOH’s reported date of the first drain and the corresponding oyster age:


Date:time of first fill=Date:timeoffirstdrain−Age at first drain (hours). (2)

This calculated date served as the temporal anchor for extracting associated environmental conditions.

### Environmental data

Environmental data were sourced from multiple platforms. To capture potential lagged effects on oyster hatchery production, environmental variables were incorporated both in their original form and with lags ranging from 1 to 7 days, and several variables representing winter conditions.

### Buoy observations

Quality-controlled records were obtained from the Goose’s Reef (GR) buoy, part of the NOAA’s Chesapeake Bay Interpretive Buoy System (CBIBS). The water quality parameters included:

- Chlorophyll concentration (*μ*g/L)
- Dissolved oxygen concentration (mg/L)
- Salinity (PSU)
- Turbidity (NTU)
- Water temperature (^°^C)

In addition to water quality, the buoy provided atmospheric data:

- Air temperature (^°^C)
- Wind direction (degrees clockwise from true north)
- Wind speed (m/s)

While investigating water quality impacts on hatchery production is an intuitive approach, including weather data was crucial for developing predictive tools and potentially an early warning system, given the influence of weather on Choptank water quality and, subsequently, hatchery production.

The following data cleaning and imputation procedures were applied to the GR buoy data:

- Only data with the highest quality codes (1 and 2) were retained.
- The original 10-minute interval data were aggregated to obtain daily averages for all variables.
- Simple linear regressions ($R^{2}>0.95$) between buoy data and corresponding measurements from NOAA’s station CAMM2 in Cambridge, Maryland, were used to impute missing daily air and water temperatures where feasible.
- Missing daily air temperatures from the GR buoy were additionally filled using a simple linear regression with air temperature data from the Cambridge airport. (Linear regressions for wind speed and direction between GR and both CAMM2 and the Cambridge airport yielded low $R^{2}$ and were therefore not used for imputation.)
- Remaining missing data for the GR buoy were imputed using the iterative random forests algorithm [7] implemented in the R package *missForest* [8]. The missForest used the random seed 123 and was set to run for up to 100 iterations. At each iteration, a random forest with 50 regression trees incorporated all available variables from the buoy, station CAMM2 (air and water temperatures, wind speed, and direction), and the Cambridge airport (air temperature, wind speed, and direction), along with numeric representations of year, month, and day of the year to leverage time-series patterns.

These steps ensured a complete dataset of environmental conditions measured near the HPLOH, encompassing both water quality and atmospheric information.

### Gridded weather conditions

Daymet (DM) provides gridded daily weather estimates derived from statistical modeling techniques that interpolate and extrapolate ground-based observations [9]. For the 1 km × 1 km grid cell corresponding to the HPLOH’s location, we obtained the following daily time series:

- Precipitation (mm)
- Maximum temperature (^°^C)
- Minimum temperature (^°^C)

The daily average temperature (^°^C) was subsequently calculated as the mean of the minimum and maximum daily temperatures. We accessed the DM Version 4 dataset using the R package *daymetr* [10].

### Remote sensing products

Google Earth Engine pipelines were utilized to collect daily time series for the following remotely sensed variables:

- Normalized Difference Vegetation Index (NDVI). NDVI measurements were obtained from both the Landsat-8 Operational Land Imager (OLI) and the Sentinel-2 MultiSpectral Instrument (MSI). As an indicator of vegetation health, NDVI was used to assess the potential influence of agricultural practices and other land uses on water quality. These measurements were aggregated across the broader Choptank River watershed and its adjacent subwatersheds. We specifically examined changes (differences) in NDVI over various preceding time intervals: 1, 2, 4, 8, 16, and 32 days. This yielded seven distinct NDVI variables, providing insights into the delayed effects of agricultural activities such as fertilizer and herbicide application.
- Chlorophyll. Remotely-sensed chlorophyll measurements were also derived [11] and averaged for the same Choptank River subwatershed used for NDVI calculations. This remotely-sensed chlorophyll variable served as an indicator of phytoplankton abundance, and consequently, water quality.

### Winter averages

To account for the potential carry-over effects of winter conditions on subsequent hatchery production seasons, we calculated the average of the following daily variables for the months of January and February of each year:

- Air temperature (GR)
- Chlorophyll concentration (GR)
- Dissolved oxygen concentration (GR)
- Salinity (GR)
- Water temperature (GR)
- Precipitation (DM)
- NDVI (remote sensing)

In total, our dataset comprised 133 predictors, including the 28 original variables (Table 1) and their various lagged versions.

**Table 1:** Summary of the response variable (yield), original predictors, and winter averages.

Variable (unit)	n	Mean	SD	Min	Max	Range	SE
Yield (%)	615	10.097	9.474	0.000	56.359	56.359	0.382
Avg shell height of females (mm)	612	114.837	10.435	82.457	187.600	105.143	0.422
Avg shell height of males (mm)	608	112.614	15.712	57.556	296.319	238.764	0.637
Fecundity (millions of eggs/female)	614	13.685	7.371	0.000	48.100	48.100	0.297
Gonadal index (1 to 4)	556	2.552	0.259	1.500	3.250	1.750	0.011
Spawn pH (NBS units)	459	7.796	0.242	6.701	8.305	1.604	0.011
Spawn salinity (PSU)	539	10.115	1.614	5.875	14.187	8.312	0.070
Spawn temperature (^°^C)	539	19.742	1.918	15.674	27.640	11.966	0.083
Week (1 to 52)	615	24.418	7.254	9.000	39.000	30.000	0.292
GR air temperature (^°^C)	615	20.645	6.709	−0.568	30.541	31.109	0.271
GR chlorophyll (*μ*g/L)	615	8.866	5.968	0.619	47.057	46.438	0.241
GR oxygen (mg/L)	615	9.505	1.905	4.772	14.635	9.863	0.077
GR salinity (PSU)	615	10.685	2.433	2.596	17.059	14.463	0.098
GR turbidity (NTU)	615	4.667	5.425	0.315	41.114	40.799	0.219
GR water temp (^°^C)	615	21.326	7.058	0.369	30.342	29.973	0.285
GR wind direction (1^°^ to 360^°^)	615	182.762	55.159	48.720	322.340	273.620	2.224
GR wind speed (m/s)	615	4.688	1.828	0.000	11.514	11.514	0.074
DM precipitation (mm/day)	615	4.641	10.383	0.000	99.230	99.230	0.419
DM temperature avg (^°^C)	615	21.231	6.560	−1.305	31.050	32.355	0.265
DM temperature max (^°^C)	615	26.276	6.535	1.620	36.510	34.890	0.264
DM temperature min (^°^C)	615	16.187	6.862	−4.260	26.180	30.440	0.277
Chlorophyll in Choptank (*μ*g/L)	615	33.865	14.721	6.790	83.061	76.271	0.594
NDVI (−1 to 1)	615	0.476	0.222	−0.023	0.929	0.953	0.009
NDVI 1-day difference	615	0.001	0.077	−0.907	0.755	1.662	0.003
NDVI 2-day difference	615	0.006	0.122	−0.895	0.846	1.742	0.005
NDVI 4-day difference	615	0.006	0.157	−0.871	0.706	1.577	0.006
NDVI 8-day difference	615	0.023	0.187	−0.824	0.884	1.708	0.008
NDVI 16-day difference	615	0.042	0.209	−0.624	0.945	1.569	0.008
NDVI 32-day difference	615	0.079	0.225	−0.879	0.726	1.605	0.009
Winter NDVI (−1 to 1)	615	0.270	0.082	0.168	0.458	0.291	0.003
Winter precipitation (mm/day)	615	3.075	0.697	1.676	4.002	2.326	0.028
Winter water temperature (^°^C)	615	3.806	1.458	1.696	6.109	4.413	0.059
Winter salinity (PSU)	615	13.657	1.496	9.669	15.619	5.950	0.060
Winter oxygen (mg/L)	615	12.817	0.391	12.343	13.602	1.259	0.016
Winter chlorophyll (*μ*g/L)	615	7.831	2.724	2.129	11.102	8.973	0.110
Winter air temperature (^°^C)	615	2.872	1.954	−0.404	5.983	6.387	0.079

### Machine-learning methods

In this study, we employed data-driven machine-learning methods to investigate the impact of various environmental and hatchery variables on the production yield and to identify key predictors. Our primary predictive model was the random forest, known for its robustness and predictive power [12–15]. To optimize its performance and select the most relevant variables, we explored several variable selection techniques and combinations of random forest hyperparameters. The chosen techniques and model parameters were validated by assessing model accuracy on deliberately held-out data using cross-validation. As alternative and complementary approaches, we developed neural network and additive statistical models. Finally, we utilized Shapley values to interpret the individual contributions of variables to model predictions and to decompose specific hatchery yield forecasts. The following subsections describe each method in detail, including the software packages used for implementation.

### Random forest

We employed random forest, an ensemble learning method that aggregates predictions from multiple decision trees to model complex, non-linear relationships. By constructing trees on bootstrap samples and considering only a random subset of predictors at each split, random forest effectively reduces variance and mitigates overfitting compared to individual decision trees.

Models were implemented using the R package *ranger* [16]. To optimize predictive performance, we performed a grid search over key hyperparameters using cross-validation (described below). We fixed the number of trees at 500, as error rates were observed to plateau beyond this count. We then systematically varied the number of variables available for splitting at each node (mtry∈{5,10,20,30}) and the minimum terminal node size (nodesize∈{1,3,5,10}). The bootstrap sample size matched the original dataset size.

### Identification of important predictors (variable selection)

Variable selection techniques were applied to improve the performance of the random forest model and, consequently, to identify the most important predictors of hatchery yield. The presence of numerous irrelevant predictors among the original inputs can reduce the likelihood of a relevant predictor being selected within the random *mtry* subset when creating a new tree split [17]. Reducing the number of irrelevant predictors helps ensure that most *mtry* subsets contain at least one relevant predictor for tree growth. However, it is not strictly necessary to eliminate all irrelevant predictors, as they may not be selected for splitting if better alternatives exist within the *mtry* set [17].

From a diverse array of variable selection methods, we applied two high-performing algorithms specifically designed for random forest models: Boruta [18] and interaction forest [19]. We implemented each algorithm in both a single pass and a recursive manner (continuing until no more irrelevant predictors were identified), comparing their performance against a baseline where all predictors were used without selection. This resulted in five distinct variable selection strategies, which were then evaluated using a cross-validation study.

The Boruta algorithm assesses predictor significance by creating permuted “shadow” copies of the original predictors. It then calculates the importance of these shadow predictors and groups their importance values (e.g., into maximal, mean, and minimal). Since shadow predictors are inherently irrelevant due to their random permutations, their importance distribution serves as a null model for irrelevant predictors. The algorithm uses a *t*-test to compare the maximal shadow importances with the importances of real predictors, with *p*-values adjusted for multiple comparisons using the Bonferroni method [18]. In our single implementation of Boruta, we retained only predictors that exhibited significantly higher importance than the maximal shadow importances. In our recursive implementation, only “rejected” predictors (those with importances significantly lower than the shadow ones) were removed at each step, and the selection process was repeated until no further rejections occurred. We used the R package *Boruta* [20] with its default settings for this algorithm.

The interaction forest algorithm introduces an effect importance measure (EIM) that facilitates ranking the importance of individual predictors (similar to traditional approaches) and also evaluates bivariate split effects (interaction effects) and their importance. In our basic interaction forest implementation, all original predictors were used without prior variable selection, meaning only the split selection differed from conventional random forests. In our recursive interaction forest implementation, predictors with a univariate EIM <0 were removed, and the interaction forest was re-trained. We also explored selection predictors with a bivariate EIM > 0 (indicating a positive importance of interaction effects); however, this approach did not lead to a reduction in the number of retained predictors. Hence, the results did not differ from the non-recursive implementations and are not reported explicitly in this study. We used the R package *diversityForest* [21] to train the interaction forests.

### Neural network

Neural networks have been widely applied to model complex non-linear relationships between variables [22–24]. Our neural network model comprised three layers: an input layer, a hidden layer, and an output layer. The hyperparameters associated with the nodes in these layers and the activation functions were optimized using the R package *kerastuneR* [25].

We evaluated the performance of numerous models with various hyperparameter combinations. This included varying the number of nodes in the input and hidden layers from 10 to 100, while the output layer consistently had 1 node. The activation function for the input and hidden layers was set to exponential linear unit (ELU), while for the output layer, we tested sigmoid, linear, softplus, and softmax functions. Furthermore, three learning rates were assessed: 0.01, 0.001, and 0.0001. A maximum of 20 trials were conducted, with 3 executions per trial. The optimal model among these configurations was selected based on the mean squared error (MSE) loss function.

The final selected neural network model comprised 141, 120, 175, and 1 nodes in the input, hidden (dense) layer 1, hidden (dense) layer 2, and output (dense) layers, respectively, with a learning rate of 0.001 and a sigmoid activation function for the output layer. The sigmoid activation function was specifically chosen for the output layer because it constrains predictions to a range between 0 and 1, aligning with the expected range of yield percentages after scaling. To accelerate model convergence, avoid the undue influence of large-valued, unimportant features, and reduce the sensitivity of activation functions [26–28], both response and predictor variables were scaled to a range of 0–1 using the R package *caret* [29]; in particular, yield percentages were converted to proportions by dividing by 100 and predictors were scaled to [0, 1] range using min-max normalization. While the neural network was trained on this scaled [0, 1] interval, all predictions were back-transformed (multiplied by 100) prior to evaluation and comparison with other models. The neural network models were trained using the R packages *keras* [30] and *tensorflow* [31]. The optimized model parameters from the best model were subsequently used for cross-validation and model interpretation.

### Generalized additive models

To assess predictive performance using a semi-parametric statistical approach, we implemented generalized additive models (GAMs). Unlike the tree-based or neural network approaches, GAMs model the relationship between the target variable and predictors as a sum of smooth functions. Given the high dimensionality of the dataset (133 variables), direct application of GAMs was computationally infeasible and prone to overfitting. Therefore, we first applied principal component analysis (PCA) to the predictor set to reduce dimensionality while retaining at least 80% of the variance.

We then fitted GAMs using the resulting principal components as predictors. To identify the most parsimonious model, we utilized stepwise selection (combining both forward and backward selection) based on the Akaike information criterion (AIC) in the R package *gamlss* [32].

### Cross-validation for performance evaluation

To compare different model configurations and variable selection strategies, and to select the best-performing option for our final model, we employed temporally-nested cross-validation. The HPLOH dataset includes years 2011–2021. All models were trained on data for years 2011–2018, 2011–2019, 2011–2020, while tested on data from 2019, 2020, and 2021, respectively. Additionally, we utilized *k*-fold cross-validation, such as for variable selection or hyperparameter tuning on the training set. In this approach, the input cases are first randomized and then partitioned into *k* equally sized folds (subsets). The model is trained using k−1 folds, and then predictions are made for the remaining single fold, which serves as the validation fold. This procedure is iterated *k* times, with each of the *k* folds serving as the validation set exactly once. We used k=10 and the R package *caret* [29] to manage the data partitioning, ensuring the same randomization was applied when comparing competing models.

The predictions generated on the validation folds and testing data from the years 2019, 2020, and 2021 are considered out-of-sample forecasts because the corresponding data were not used during the model specification or training phases. Let $e_{i}$ denote the cross-validation error for the *i*-th observation, defined as the difference between the true yield ($y_{i}$) and the forecast ($\hat{y_{i}}$):


$$e_{i}=y_{i}-\hat{y_{i}},$$ (3)

where i=1,2,…,n and *n* is the total sample size. We evaluated the performance of alternative models using three key metrics: mean absolute error (MAE) and root mean square error (RMSE), which were calculated as follows:


$$MAE=n^{-1}\sum |e_{i}|,$$ (4)

$$RMSE={\left(n^{-1}\sum e_{i}^{2}\right)}^{1/2},$$ (5)

where $\bar{y}=n^{-1}\sum y_{i}$ is the average observed yield. Smaller MAE and RMSE are preferred, with RMSE penalizing larger individual errors ($e_{i}$) more heavily than MAE. While MAE and RMSE generally provided consistent performance insights, RMSE was ultimately used for making the final model selections.

### Model interpretation

Model interpretation techniques are crucial for enhancing the understanding of “black-box” models by providing insights into the complex relationships learned and identifying which input variables exert the most influence on predictions. While partial dependence plots are a common way to visualize pairwise predictor-response relationships captured by a random forest [17], they do not offer explanations for individual predictions. In this study, our primary method for interpretation was Shapley decomposition, which explains the contribution of individual predictors [33]. We specifically utilized fast (approximate) Shapley values to explain individual predictions in tree-based models like random forests [34]. Furthermore, we also computed conventional partial dependence plots to validate the insights gained from the Shapley values of individual predictors.

The SHAP (SHapley Additive exPlanations [35]) values $\phi_{j}$ possess an additivity property, allowing the forecast of hatchery yield for a given vector of inputs *x* to be decomposed as a sum [33]:


$$\hat{y}(x)=\phi_{0}+\sum_{j=1}^{p}\phi_{j},$$ (6)

where $\hat{y}(x)$ is the forecast, $\phi_{0}=E(\hat{y}(X))$ represents the baseline value (set to the average prediction across all cases), and *p* is the number of predictors in the model.

We employed SHAP values in several ways for model interpretation:

- First, we ranked the importance of predictors using the average absolute SHAP values (denoted as mean(|SHAP value|) in figures). This metric is analogous to the permutation-based importance in random forests, indicating the average impact of each variable on predictions across the dataset.
- Second, we summarized the explained effects for specific subsets of cases. We were particularly interested in well-predicted cases (i.e., those where the model accuracy was high, with an absolute error below 3 percentage points). To analyze the environmental and hatchery conditions contributing to exceptionally low or high yields, we selected such events that were well-predicted by our models.
- Third, we aggregated the Shapley values within groups to avoid multicollinearity issues and redundancy in the predictors representation (Table 2). Multicollinearity among environmental predictors can complicate model interpretation, as the "credit" for a prediction may be distributed among correlated features, effectively diluting their individual Shapley values. The grouping and aggregation of Shapley values was consistently applied to both random forest and neural network models to ensure that the cumulative contribution of correlated drivers was accurately captured and that high-ranking variables represented genuine environmental signals.

**Table 2:** Groups of variables for aggregating SHAP values.

Category	Variables
AvgShellHeight	Avg shell height of females, Avg shell height of males
Air Temperature	GR air temperature, GR air temperature lag1–lag7, DM temperature avg, DM temperature avg lag1–lag7, DM temperature max, DM temperature max lag1–lag7, DM temperature min, DM temperature min lag1–lag7, Winter air temperature
Chlorophyll	GR chlorophyll, GR chlorophyll lag1–lag7, Winter chlorophyll
Oxygen	GR oxygen, GR oxygen lag1–lag7, Winter oxygen
Salinity	GR salinity, GR salinity lag1–lag7, Winter salinity, Spawn salinity
GR Turbidity	GR turbidity, GR turbidity lag1–lag7
Water Temperature	GR water temperature, GR water temperature lag1–lag7, Winter water temperature, Spawn temperature
WindDirection	GR wind direction, GR wind direction lag1–lag7
WindSpeed	GR wind speed, GR wind speed lag1–lag7
Precipitation	DM precipitation, DM precipitation lag1–lag7, Winter precipitation
Chrolophyll in Choptank	CHLct, CHLct lag1–lag7
NDVI	NDVI, Winter NDVI, NDVI d1, NDVI d2, NDVI d4, NDVI d8, NDVI d16, NDVI d32, NDVI lag1–lag7

To compute Shapley additive explanations for random forest models, we used the R package *fastshap* [36]. For neural networks, the R package *reticulate* [37] served as a wrapper to extract SHAP values. In both cases, the *shapviz* [38] package was used for visualization.

## Results

### Model and variable selection

**Fig 1:**
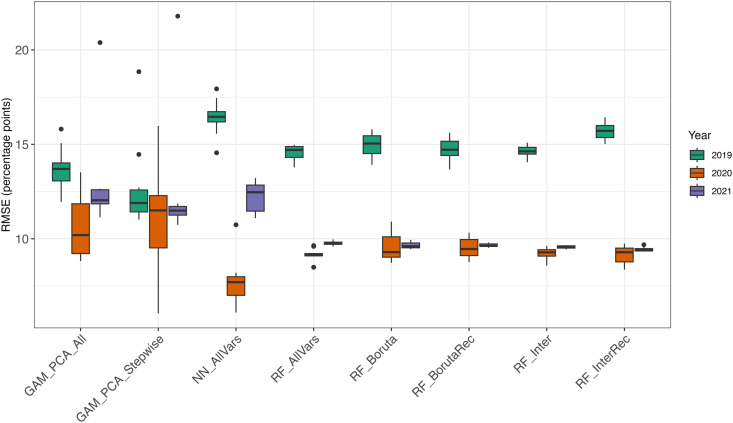
Model performance. Root mean square error (RMSE) from cross-validation evaluating alternative model options. Here, acronyms are - GAM_PCA_All: Generalized Additive Model (GAM) fitted using all derived principal components, GAM_PCA_Stepwise: GAM fitted using principal components with stepwise model selection, NN_AllVars: Neural Network using all predictor variables, RF_AllVars: Random Forest using all predictor variables, RF_Boruta: Random Forest with feature selection using the Boruta algorithm, RF_BorutaRec: Random Forest with recursive Boruta feature selection, RF_Inter: Random Forest with interaction-based feature selection, and RF_InterRec: Random Forest with recursive interaction-based feature selection. Each boxplot represents the predictive performance of the respective model on the test set.

Our evaluation of variable selection algorithms—Boruta, recursive Boruta, and recursive interaction forest—demonstrated only slight improvements in model performance compared to using all available variables in the random forest model or all numeric variables in the neural network model. Specifically, these selection methods led to lower errors compared to other models (Fig 1 for RMSE and S1 Fig for MAE).

Recursive Boruta generally selected fewer variables than the recursive interaction forest but more than the non-recursive Boruta (S2 Fig). The non-recursive interaction forest, by design, used all variables.

Given these findings, we chose random forest with all variables as the best-performing model. However, we also see the value in reducing the number of predictors, such as with the Boruta algorithm. When applied to the entire dataset, recursive Boruta identified 55 predictors for further analysis. This suggests that not all numeric predictors are equally critical for yield predictions.

### Model summaries

The selected random forest model trained on the whole dataset achieved an out-of-bag MAE of 6.0 percentage points and an RMSE of 7.9 percentage points. The optimal model, identified by achieving the lowest RMSE in cross-validation, used an *mtry* of 30 randomly selected predictors at each tree split and a *nodesize* of 10 observations. The average predicted yield from this model (9.7%) closely aligned with the average observed yield (9.6%) in the dataset.

Conversely, the best-performing neural network model produced an average predicted yield that was slightly higher (10.4%) than the random forest models. The neural network showed comparable performance in cross-validation, with an MAE of 9.9 percentage points and an RMSE of 12.1 percentage points.

## Shapley value analysis

### Global insights

**Fig 2:**
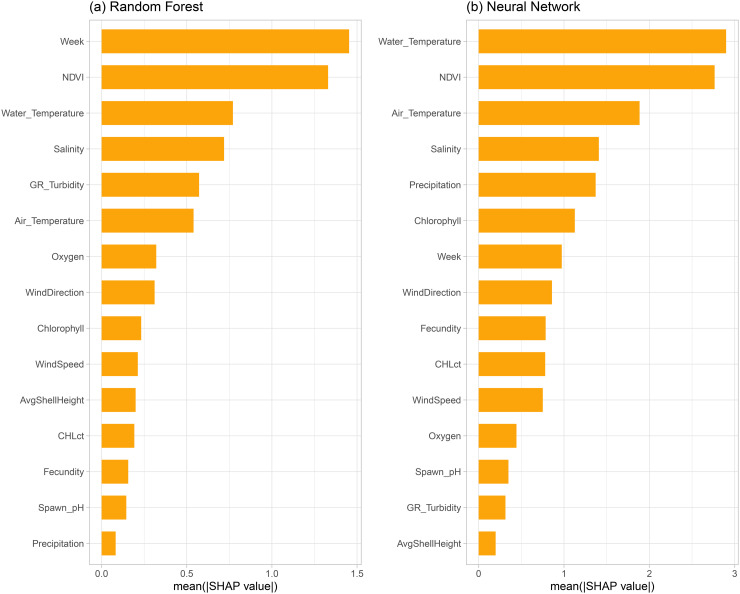
Feature importance. Shapley-based feature importance scores for the models estimated on the whole dataset (higher values correspond to more important variables): **(a)** Random forest, and **(b)** Neural network. The horizontal axis shows average absolute impact of each predictor on the model output (hatchery yield, %). Only the most important predictors are shown.

Shapley values offered crucial insights into our models by identifying key predictors. Fig 2 illustrates the average magnitude of variable effects for both random forest and neural network models. For the random forest, week number, NDVI, water temperature, salinity, and turbidity emerged as the top determinants of hatchery yields. While water temperature, NDVI, air temperature, and salinity were also prominent in the neural network, other important variables included precipitation-related terms and winter chlorophyll. Water temperature exerted a greater influence on predictions in the neural network model, while week number was ranked 7th (Fig 2).

Although the ranking and importance of variables differ between the random forest and neural network models, the same variables are the top predictors in these two models. In the random forest model, week number and NDVI are the two most important variables, with mean SHAP values above 1 and substantially higher than for other variables. The neural network model also ranks NDVI as a top variable, water temperature, air temperature, salinity, precipitation and chlorophyll as the most important variables with observed mean SHAP values above 1, while week number drops to 7th place with a lower mean SHAP value. While both models identify wind direction and fecundity as important, their relative rankings differ, with wind direction being at the same level (8th), and fecundity being the 13th in the random forest and 9th in the neural network. This highlights how the choice of model architecture can lead to different interpretations of variable importance, even when trained on the same data.

### Insights into low yields

**Fig 3:**
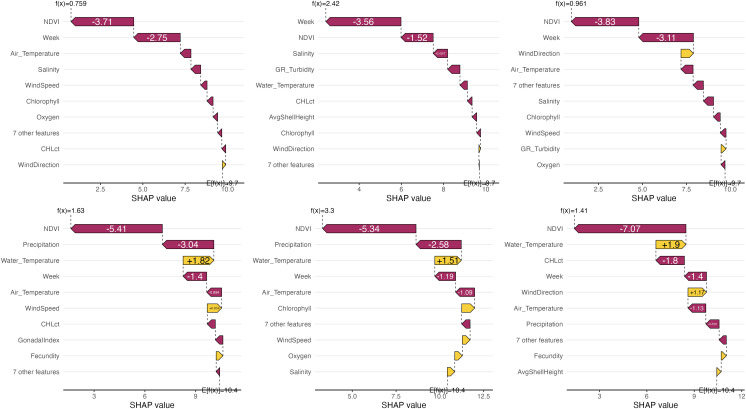
Contributions of predictors to well-predicted low-yield cases. Individual predictor contributions to three of the cases are shown for the random forest model (top row) and the neural network model (bottom row). The baseline E[f(x)] represents the average predicted value across all dataset cases, while f(x) is the predicted value for the specific case. Arrows indicate the largest impacts of individual and grouped predictors, with less influential predictors aggregated.

We further analyzed model insights specifically for low hatchery yields. We identified 73 well-defined low-yield cases (cases with yields below 1% and absolute model errors below 3 percentage points) from the random forest predictions, and 20 from the neural network. Between these cases, 18 were common to both models, from which we selected three examples illustrated in Fig 3. For these specific cases, the random forest model still highlighted week and NDVI as the two most important variables with the largest absolute contributions. However, the subsequent five important variables were related to air temperature, salinity, wind speed, chlorophyll and oxygen. In the neural network model, NDVI and precipitation were found as more critically contributing to the low-yield cases (Fig 3). Both these variables also showed strong overall influence on yield (Fig 2).

### Insights into high yields

**Fig 4:**
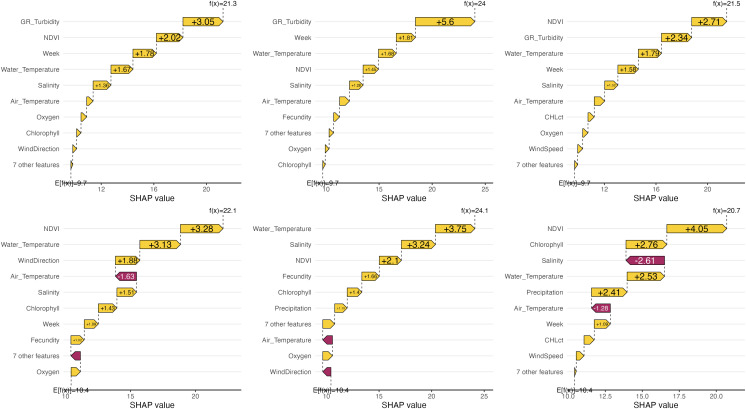
Contributions of predictors to well-predicted high-yield cases. Individual predictor contributions to three of the cases are shown for the random forest model (top row) and the neural network model (bottom row). The baseline E[f(x)] represents the average predicted value across all dataset cases, while f(x) is the predicted value for the specific case. Arrows indicate the largest impacts of individual and grouped predictors, with less influential predictors aggregated.

Similarly, we investigated cases of well-defined high yields common between both models, selecting 3 instances where yields were above 20% and the absolute model error was below 3 percentage points. For these high-yield cases, the ranking of influential variables (Fig 4) appeared comparable to the global rankings (Fig 2). In the random forest predictions, turbidity, NDVI, and week variables are important, alongside salinity and water temperature. This shift may be attributed to these high-yield cases typically occurring during a favorable season, with weeks ranging from 15 to 29 (average 23) and lower winter NDVI values (0.22 to 0.26, average 0.24). Contrarily, neural network showed that NDVI, water temperature, and chlorophyll contributed dominantly to yields. Additionally, these variables had the highest overall importance values for the whole dataset. Similarly to random forest, in high-yield cases, salinity was highlighted as one of the primary factors.

### Relationships between predictors and yield

To better understand the factors influencing oyster production, we created partial dependence plots for the top 10 predictors identified by the SHAP values (Fig 2). We grouped partial dependence plots for consistency following the same categories as in Table 2. These plots reveal how each variable affects hatchery yield.

The random forest model showed that as the growing season progressed (indicated by a higher week number), yield tended to decrease (S3 Fig). Similarly, higher air temperature, water turbidity and NDVI were all associated with lower yields. In contrast, yield increased with higher salinity, wind speed, NDVI, and water temperature.

The neural network model largely agreed with these findings (S4 Fig). Its partial dependence plots also showed that increased NDVI and air temperature led to lower yields, along with higher winter chlorophyll and precipitation. The factors positively associated with yield were salinity and water temperature. Except for chlorophyll, which appears plateaued after 10 μg/L in both models’ partial dependence plots but showing negative association in neural network and positive in random forest.

Overall, both models captured similar relationships, highlighting the key associations behind either a decrease or an increase in hatchery yield.

## Discussion and conclusion

### Model performance and robustness

Our study leveraged advanced machine-learning techniques to unravel the complex factors influencing oyster hatchery production yield, addressing a critical need within Maryland’s oyster aquaculture industry. Driven by the important economic and ecological role of oysters in the Chesapeake Bay, our work aimed to apply predictive modeling and interpretation to enhance hatchery resilience and efficiency. The model performance was comparable in temporally-nested cross-validation folds except for year 2019 which showed anomalous behavior during testing possibly related to the smaller sample size than in the other test years. In addition, to prevent model overfitting, we prioritized robust feature selection and tuning the model hyperparameters. We found that random forest models, particularly when coupled with robust variable selection methods like recursive Boruta, provide accurate out-of-sample predictions of hatchery yields. The neural network model, despite using a large suite of predictors, demonstrated comparable predictive performance with random forest. It showed a high variability in RMSE for the three testing years, compared to the random forest results (except for year 2019 when all the models performed poorly). 

### Key predictors of hatchery yield

Our findings offer several takeaways for oyster aquaculture. Global Shapley value analysis revealed that week, NDVI, water temperature, salinity, and turbidity are among the most influential determinants of hatchery yields in random forest models. While some overlap exists, the neural network highlighted precipitation and air temperature as additional dominant variables.

By analyzing SHAP values for specific scenarios, we gained nuanced insights. In well-predicted low-yield events, besides week and NDVI, water salinity-related variables, wind direction and air temperature became particularly prominent. Conversely, high-yield events showed increased importance for various turbidity indicators, alongside fecundity, NDVI, and oxygen, reflecting the favorable conditions during these periods.

Multicollinearity of predictors was addressed by grouping correlated predictors during the Shapley and partial dependence plot analysis. This confirmed that the high importance of variables such as NDVI and salinity reflects a robust association with hatchery yield.

Short- and long-term salinity fluctuations have repeatedly influenced production at HPLOH, but previous analyses demonstrated that this relationship is more complex than a simple salt concentration effect. For example, adding artificial salts during low-salinity periods did not restore production [1]. We hypothesize that either (a) freshwater inflow delivers additional stressors from upstream or adjacent land uses, such as pesticide residues, or (b) low salinity alters microbial and phytoplankton communities such that the incoming water becomes biologically unfavorable for larval development. Recent work at HPLOH supports this view, showing that low-yield larval batches exhibit distinct microbial assemblages, including proliferation of opportunistic taxa and potentially harmful microeukaryotes, relative to healthy batches [39].

NDVI has proven useful as a remote proxy for terrestrial vegetation cover, crop activity, and even pesticide application intensity, which can now be monitored from space [40]. The link between NDVI and hatchery performance likely reflects watershed-scale processes that influence the quality of source water entering the hatchery. For instance, [41] detected diverse pesticide residues—including herbicides and fungicides—in estuarine oysters downstream of agricultural catchments, while [42] observed physiological stress and reproductive disruption in oysters exposed to low, environmentally realistic herbicide concentrations. Although we do not suggest that such processes are occurring in the Choptank River, these examples illustrate how land use and pesticide runoff can alter microbial ecology, water quality, and oyster health in coastal systems.

Our finding that NDVI correlates with hatchery performance thus underscores the potential of using integrative, landscape-scale indicators to capture linkages between terrestrial activity and aquatic outcomes. However, we emphasize that the relationship observed here is correlative rather than mechanistic—a signal that merits deeper investigation. Establishing causality will require targeted measurements of nutrients, organic matter, and contaminant loads in conjunction with microbial and physiological monitoring of hatchery water. Nevertheless, this represents a step toward connecting machine learning-based production analyses with remote sensing and land-use data, supporting a broader goal of aligning sustainable terrestrial and aquatic farming practices.

### Implications for hatchery management

Despite the intricate nature of biological and environmental interactions, our machine-learning models effectively predict hatchery outcomes based on diverse input data, including water quality, weather records, and satellite-derived greenness data (NDVI). This predictive capability provides a powerful tool for proactive management.

We anticipate this study will benefit oyster hatcheries by enabling more data-driven decision-making. Our machine-learning models can serve as an early warning system for potential cases of suboptimal larval growth. This foresight allows hatchery operators to implement proactive and preventive measures, minimizing revenue losses and operational disruptions. While the current predictive accuracy suggests the model should function as a decision-support tool rather than a fully autonomous controller, it provides a critical layer of risk assessment that was previously unavailable. Ultimately, by reducing inefficiencies and improving the predictability of yields, our research contributes to more stable and profitable operations for hatcheries. This, in turn, strengthens the economic well-being of local communities dependent on the oyster industry.

### Broader applicability of findings

While our study utilized data from a single Maryland hatchery, the implications extend beyond the Chesapeake Bay. First, the HPLOH employs standard industry protocols for broodstock conditioning and larval culture, meaning the biological and operational processes modeled here are representative of widespread hatchery practices. Second, the facility operates in a mesohaline environment that frequently approaches the lower salinity threshold for *C. virginica* production. Consequently, this study serves as a valuable test case for managing production under marginal environmental conditions—a scenario likely to become more common globally as hatcheries face increasing freshwater variability due to climate change.

Most importantly, the core methodology—leveraging machine learning for predictive modeling and SHAP analysis for interpretation—is broadly transferable. Although specific environmental thresholds will vary geographically, the analytical approach for identifying key predictors remains highly applicable. By adopting this framework, other facilities can move beyond generic best practices to identify the specific, localized predictors of their production yields.

To adapt this methodology, a new hatchery would first need to provide a comparable longitudinal dataset encompassing operational logs, biological metrics, and local water quality. This can be augmented, if needed, with publicly available environmental data (e.g., weather and remote sensing records) specific to the hatchery operation schedule. Next, the entire pipeline of model training, feature selection, and SHAP analysis should be implemented on this new dataset. The key predictors and model accuracy will be compared to those from the current analysis. This process would validate our framework’s ability to identify the most critical drivers in a new setting.

For the HPLOH that provided our data, these models can be seamlessly integrated into an early warning system. Because the hatchery already collects many of the necessary data points—such as water quality, weather, and operational metrics—it can use the models to predict yields and then immediately explore these predictions using Shapley plots. This allows staff to not only see a potential decrease in predicted yield but also to instantly understand which specific factors are driving that negative prediction, enabling them to make targeted, data-driven interventions.

Beyond HPLOH, other hatcheries must recognize the critical need to scale up their data collection efforts to use these models and eventually the hatchery resources effectively. While our study was built on data from a single location, the underlying methodology is universally applicable. Hatcheries should systematically collect a broader array of data, including detailed environmental parameters, operational logs, and biological metrics. By adopting more rigorous, standardized data collection practices, they will create the foundational datasets necessary to train and deploy similar predictive models. This will allow them to leverage the power of machine learning, moving from reactive to proactive management of their operations.

Translating predictive models into operational early warning systems will require addressing several practical challenges related to implementation and integration. Reliable environmental sensing remains a key constraint, as hatcheries differ widely in system design and access to continuous monitoring infrastructure; ensuring data quality will depend on consistent calibration, redundancy, and compatibility with automated logging. Additionally, computational capacity varies greatly across facilities, and many hatcheries operate in coastal areas with limited internet connectivity, emphasizing the need for lightweight, locally deployable models that can function independently of cloud services. Furthermore, successful adoption will depend on staff familiarity with data-driven tools and confidence in interpreting model outputs. Developing interfaces that communicate risk intuitively and incorporating basic data management and visualization training into routine operations will help bridge this gap. As a partial solution, public monitoring tools such as the National Data Buoy Center (NOAA) network can supplement local measurements, providing high-frequency oceanographic and meteorological data that capture regional trends in temperature, salinity, and wind-driven mixing. Leveraging these open datasets, along with satellite-derived and other remotely sensed products, offers a viable pathway to fill data gaps for facilities like HPLOH that routinely collect only a limited set of water quality parameters. Ultimately, the broader challenge lies not in technical feasibility but in fostering sustained collaboration between hatchery practitioners, data scientists, and engineers to build predictive systems that are accessible, interpretable, and resilient to the operational realities of shellfish production.

## Limitations and future directions

Future research on hatchery production efficiency can potentially utilize an even wider and longer array of environmental and operational parameters, such as more detailed biological metrics or genetic data of oyster stocks, could reveal additional subtle influences. A longer time series of data would facilitate the analysis of more long-term trends and a deeper understanding of climate variability’s impact over extended periods. While our study used observational data, integrating findings from controlled experimental settings (e.g., lab experiments manipulating specific parameters identified by our models) could help establish more definitive causal linkages rather than correlations. By providing actionable insights and a robust predictive framework, this study contributes to the sustainable management and resilience of oyster aquaculture, reinforcing its critical role in both the economy and ecology of coastal regions.

## Supporting information

S1 Fig Mean absolute error (MAE) from cross-validation. (PNG)

S2 Fig Number of predictors used in cross-validation. (PNG)

S3 Fig Partial dependence plots for the most important variables in the random forest model. (PNG)

S4 Fig Partial dependence plots for the most important variables in the neural network model. For training the neural network, the predictors were rescaled to the range from 0 to 1. Then, the values are back-transformed for the plots.
(PNG)

S5 Fig Feature correlations. The colorbar shows correlations: positive correlations are in red, while negative correlations are shown in blue.
(JPEG)
