# Supplementary figures and images for: Machine learning of factors for improving oyster hatchery production
Source: PLoS One. 2026 Mar 20;21(3):e0345084. doi: 10.1371/journal.pone.0345084 (PMC13004496; doi:10.1371/journal.pone.0345084)

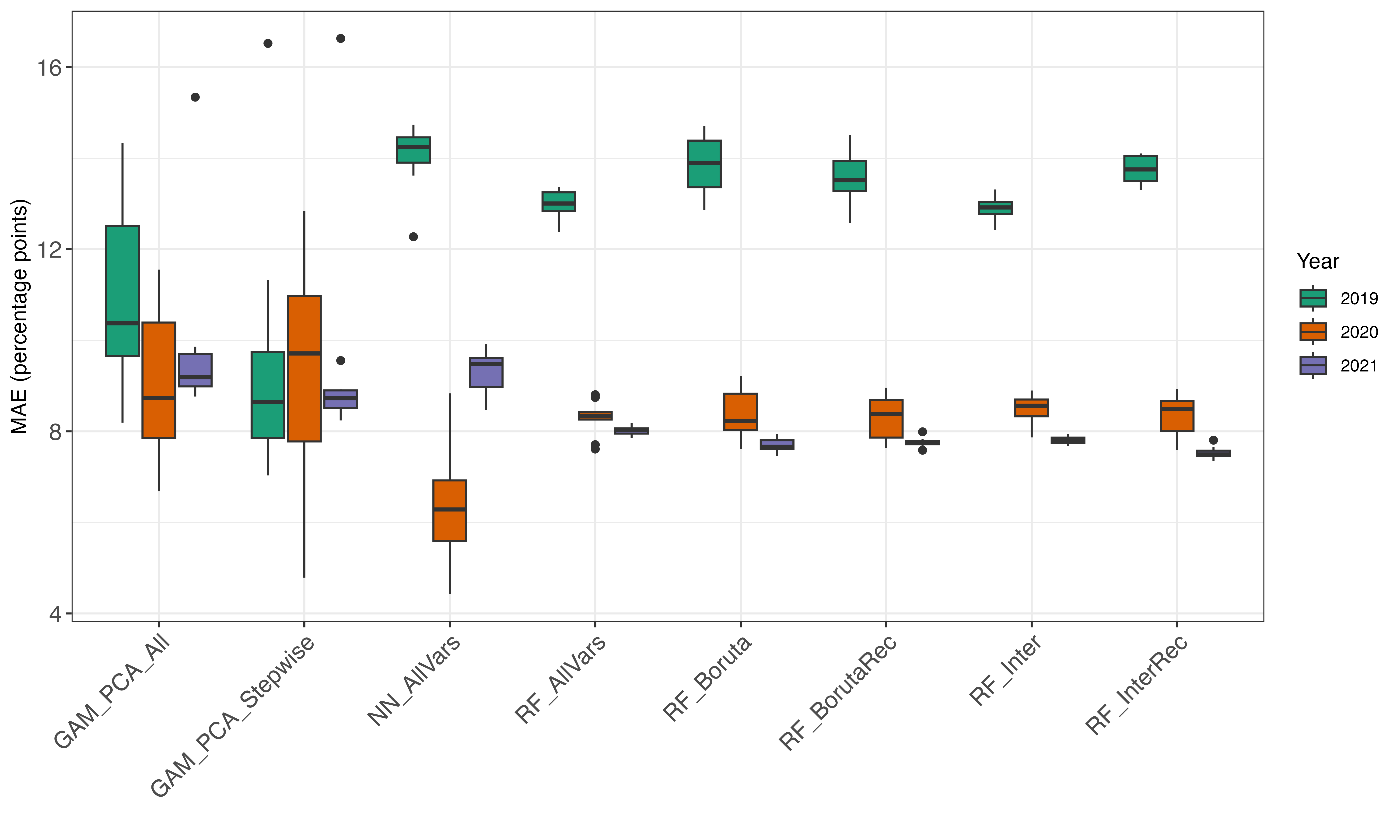

Supplement: S1 Fig — (PNG) [file pone.0345084.s001.png]

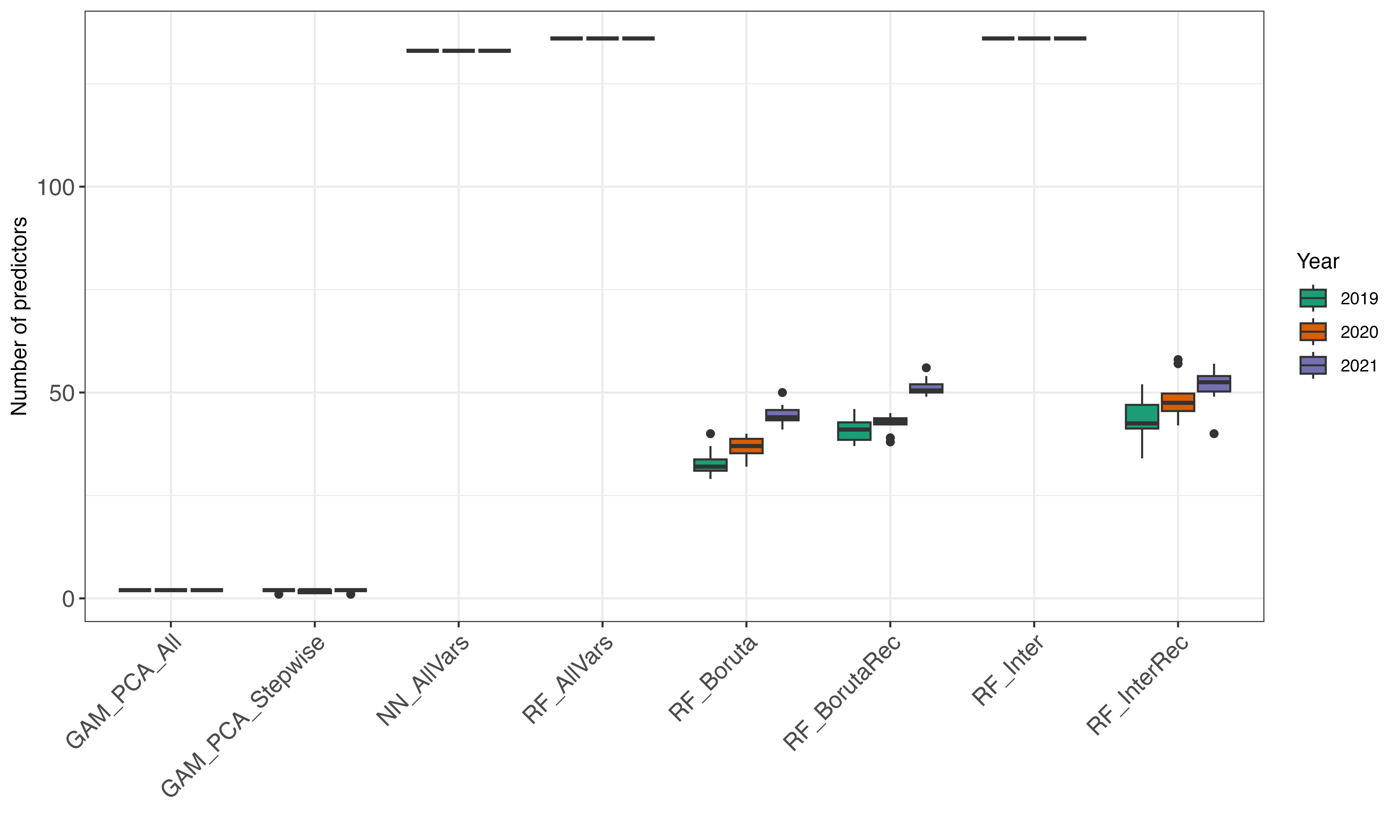

Supplement: S2 Fig — (PNG) [file pone.0345084.s002.png]

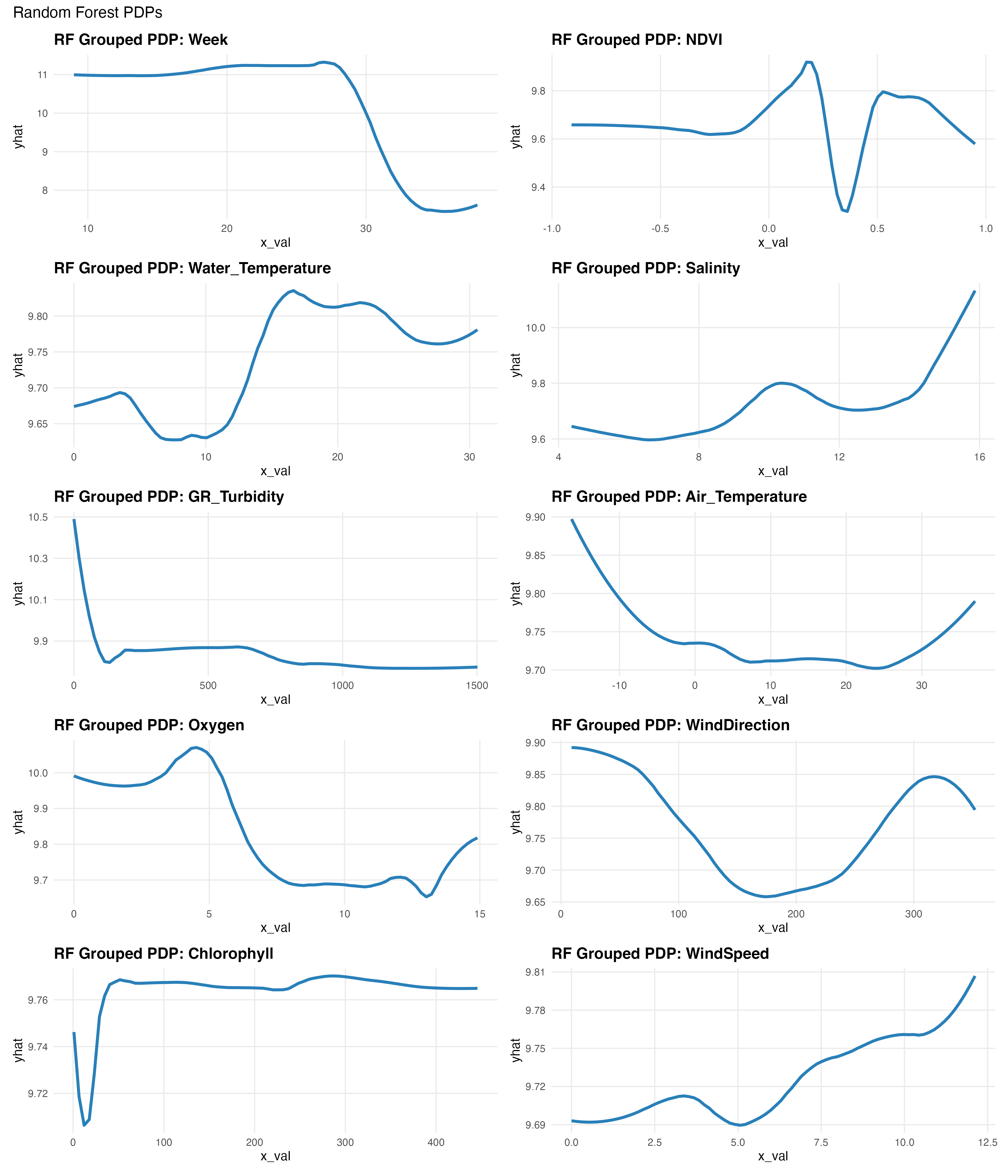

Supplement: S3 Fig — (PNG) [file pone.0345084.s003.png]

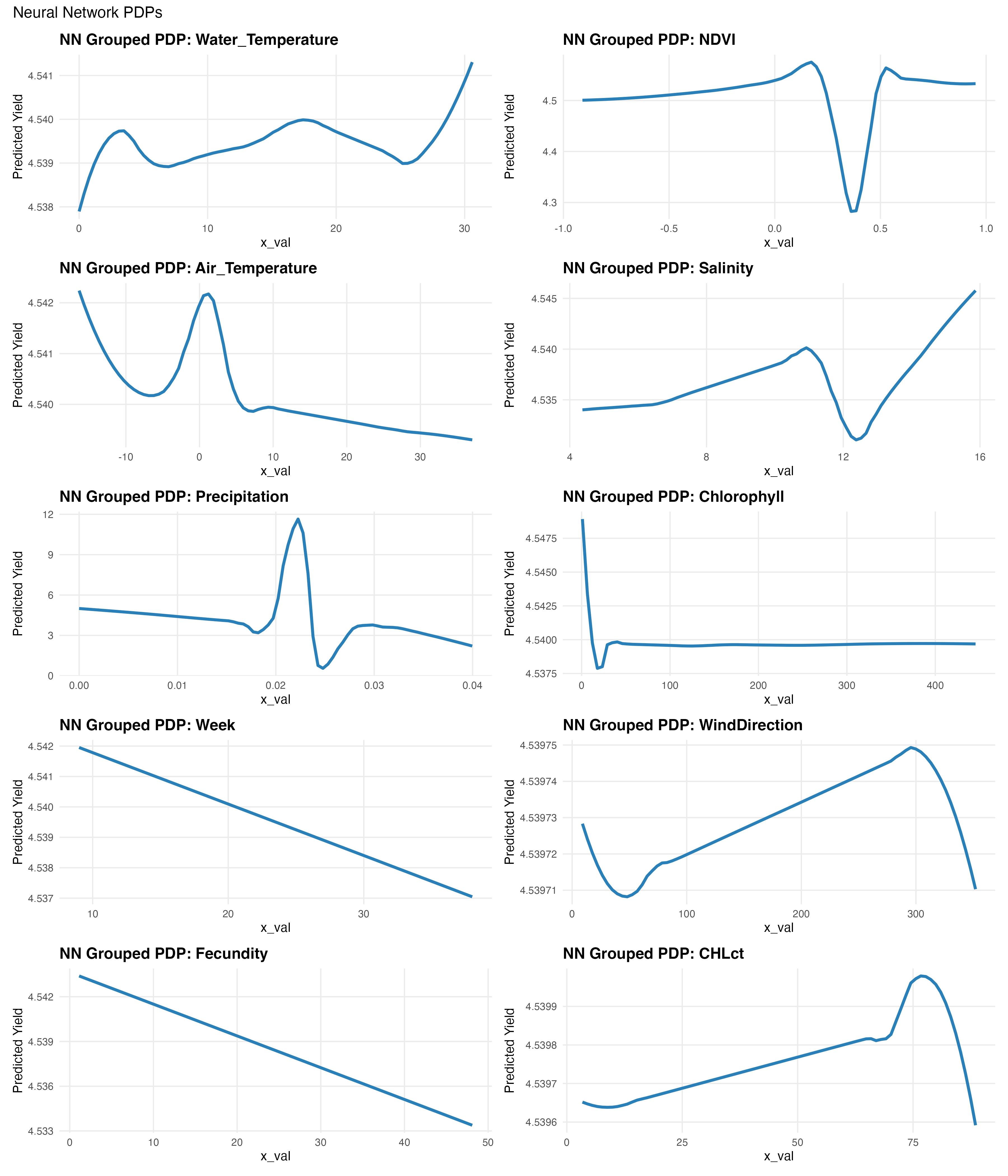

Supplement: S4 Fig — For training the neural network, the predictors were rescaled to the range from 0 to 1. Then, the values are back-transformed for the plots. (PNG) [file pone.0345084.s004.png]

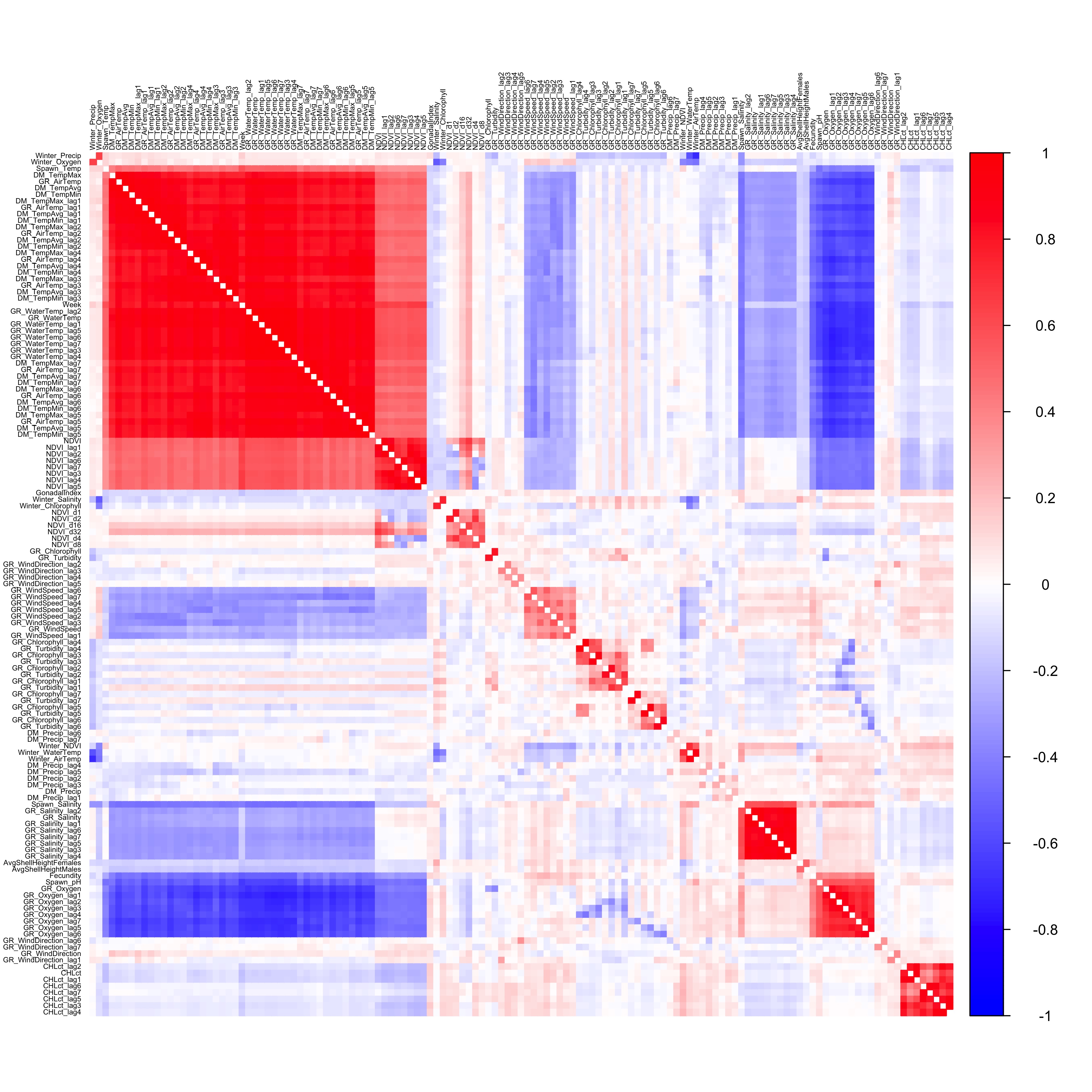

Supplement: S5 Fig — The colorbar shows correlations: positive correlations are in red, while negative correlations are shown in blue. (JPEG) [file pone.0345084.s005.jpeg]
